# Promoter Hypermethylation of the BRCA1 Gene as a Novel Biomarker for Prostate Cancer

**DOI:** 10.7759/cureus.66467

**Published:** 2024-08-08

**Authors:** Mohammed A Saleem, Mustafa S Mustafa

**Affiliations:** 1 Department of Biology, College of Science, Salahaddin University-Erbil, Erbil, IRQ

**Keywords:** gene expression, repair genes, brca1, promoter methylation, prostate cancer

## Abstract

Prostate cancer (PCa) is recognized as one of the most common malignancies that greatly affects the male population globally. Breast cancer gene 1 (*BRCA1*) is an important tumor suppressor gene that plays a central role in the maintenance of genomic integrity by promoting the repair of double-strand breaks of DNA. Here, we present a pilot study to examine the promoter methylation and gene expression of the *BRCA1* gene in patients with PCa in Erbil governorate, Iraq. The collection of samples took place in Erbil City, Iraq, specifically at Rizgary Hospital, PAR Hospital, and Al-Mufti's private laboratory. A total of 40 tissue samples were collected from age-matched individuals, comprising 30 pathologically confirmed PCa cases and 10 normal prostatic tissue taken from individuals who, during diagnosis, were found to be negative for PCa. Data on demographic and clinical information, such as pathological stage, age, and prostate-specific antigen (PSA) level, were gathered from the medical records. The impact of the promoter methylation was forecasted using the DNA bisulfite conversion technique and methyl-specific PCR (MSP) with specific primers for the *BRCA1* promoter region. The assessment of *BRCA1* expression was conducted using quantitative real-time PCR (qPCR). Among the 30 patients examined, 76.6% (23 cases) were found to have *BRCA1* promoter methylation, and none of the normal tissues appeared to have DNA methylation. *BRCA1* promoter methylation was positively associated with the advanced stage of disease (p=0.01) and Gleason score (p=0.007). The analysis revealed a significant downregulation of the *BRCA1* gene expression in methylated tumor samples as compared to non-methylated tumors and normal tissues, suggesting the role of epigenetic silencing. To the best of our knowledge, this is the first study investigating methylation status and level of *BRCA1* mRNA transcripts among PCa patients in Iraq. Our findings suggest that promoter hypermethylation of the *BRCA1* gene could serve as a viable biomarker for PCa, marking a significant discovery.

## Introduction

The molecular mechanisms driving cancerogenesis and tumorigenesis are intricately linked to epigenetic changes in gene regulation, including DNA methylation, non-coding RNAs, chromatin remodeling, and histone modification, alongside DNA mutations. Therefore, the examination of epigenetic markers for diagnosing, predicting outcomes, and preventing cancer is of significant interest to the field of medicine [1,2]. The use of DNA methylation level detection has recently emerged as a valuable biomarker for early diagnosis, prognosis, and identification of target genes for drug therapy [3,4].

The etiology of cancer, specifically the non-mutagenic causes such as DNA methylation disturbances, is influenced by various factors such as age, diet, environmental, transplacental, and occupational exposure. Due to its dual nature of being both time-reversible and transgenerational, a specific methodology must be applied when interpreting the results. Age-related DNA methylation has been linked to a range of diseases, while the influence of sex hormone status on DNA methylation has been observed in specific cancer types [5,6]. There is a substantial knowledge gap regarding the association between age-expected DNA methylation levels and cancer [7].

The initiation and progression of carcinogenesis heavily rely on epigenetic changes and modifications [8]. Aberrant epigenetic programs, such as DNA methylation, have the potential to deactivate extensive gene clusters. Tumor tissues may harbor a significant number of epigenetically silenced genes [9]. Extensive research has been carried out on DNA methylation, revealing a correlation between hypermethylation and the silencing of tumor suppressor genes in PCa, as well as poor clinical outcomes [8,10-12]. Numerous genes were found to be differentially methylated in PCa compared to adjacent normal tissues, as reported by these studies. The majority of these studies employed a candidate gene approach to enhance the statistical efficiency of the association analysis, while simultaneously limiting the assessment of methylation effects associated with multiple genes. Multiple studies utilized an epigenome-wide methylation microarray to encompass a broad spectrum of genes. All studies have reported a considerable amount of differentially methylated CpGs, yet these results remain unverified in independent validation datasets [13-15]. Considering the potential impact of DNA methylation alterations on the disparities in PCa among different racial and ethnic groups [16], it is necessary to investigate these patterns in diverse small cohorts to identify potential candidates for subsequent representative validation studies.

The identification of prostate-specific antigen (PSA) has proven to be a groundbreaking discovery in PCa. There has been a rise in excessive diagnosis and treatment of PCa in recent decades. Despite potential alterations to the standard PSA testing, a notable lack of specificity has been observed. Advances in various fields have contributed to the improvement of our understanding of PCa genetics and proteomics. The discovery of novel serum, urine, and tissue biomarkers has facilitated the development of multiple diagnostic tests. These tests effectively minimize the occurrence of unnecessary and repetitive biopsies [17].

The diagnosis of PCa is primarily determined through a pathologist's assessment of biopsied tissue, resulting in the determination of a Gleason score for the severity of the disease. This is linked to an average error rate of 25-30% for underdiagnosis and an average error rate of 1.3-7.1% for overdiagnosis [18-20]. The Gleason score is estimated to have an accuracy of 61% [21,22]. Despite the typically indolent nature of PCa, approximately 20-30% of cases display an aggressive phenotype, posing a risk of metastasis and fatality. This aggressive phenotype is characterized by the dysregulation of DNA repair genes, which is a crucial molecular feature. The targeting of DNA repair pathway defects in PCa can be achieved using PARP1 inhibitors. These drugs have been found to be particularly effective in treating prostate tumors that have defects in the DNA repair genes such as *BRCA1 *or *BRCA2 *[23,24]. Despite the existing research on genetic modifications in prostate tumors [25,26], the understanding of epigenetic control of DNA repair genes in PCa remains limited. Currently, there is a lack of published data on DNA methylation in PCa patients from the Kurdistan region of Iraq.

To the best of our knowledge, there has been no investigation into the epigenetic status of the *BRCA1 *gene in patients with PCa from Iraq. Therefore, the aim of this study was to examine the promoter methylation of the *BRCA1* gene in patients with PCa from the population of North Iraq.

## Materials and methods

Patient and sample collection

Tissue samples were collected from Rizgary Hospital, PAR Hospital, and the private Mufti laboratory in Erbil City, KRG-Iraq. This study received authorization and approval from the Human Ethics Committee of Salahaddin University-Erbil, College of Science (No: 4S/494; date: September 30, 2021; Erbil, Iraq) and was accomplished under the principles outlined in the Helsinki Declaration. A total of 40 tissue samples were examined, comprising 30 pathologically confirmed PCa cases and 10 normal prostatic tissue samples taken from individuals who, during diagnosis, were found to be negative for PCa. The samples were categorized based on the grade of PCa, Gleason score, patient age, and clinical characteristics. Formalin-fixed paraffin-embedded (FFPE) samples were collected from October 2021 to October 2023.

Inclusion criteria

Patients were selected at the time of their initial diagnosis by the oncologist. None of the cases had comorbidities, and all patients and controls were over 50 years old.

Exclusion criteria

The study excluded patients undergoing chemotherapy and those with concurrent medical illnesses.

Extraction of DNA

Genomic DNA extraction was performed using FFPE blocks, which were histopathologically verified to contain malignant PCa tumors with a volume exceeding 60%. The extraction procedure was carried out using the GeneRead DNA FFPE Kit (Cat. No. 180134, Germany) from QIAGEN, specifically engineered for the purification of DNA from FFPE tissue sections, following the guidelines provided by the manufacturer. The DNA concentrations in ng/μL and their purity, as determined by the A260/A280 ratio, were assessed using the Nanodrop spectrophotometer (Biometrics; OneDrop TOUCH).

Bisulfite modification and MSP

The purified DNA samples were subjected to bisulfite conversion using the EpiTect bisulfite conversion kit (Cat. No. 59104, QIAGEN, Hilden, Germany), following the manufacturer's protocol. Methyl-specific PCR (MSP) was conducted using primers designed for the detection of methylated and unmethylated DNA regions of *BRCA1*. The primers were validated for specificity and efficiency using multiple methods. Specificity was verified by testing primers against non-target templates. Efficiency was determined using a standard curve from DNA dilutions, ensuring consistent results. A 75 base pair PCR product was achieved by utilizing the methylated template primers: 5’-TCGTGGTAACGGAAAAGCGC-3’ (forward) and 5’-AAATCTCAACGAACTCACGCCG-3’ (reverse). The unmethylated template primers were as follows: forward primer 5’-TTGGTTTTTGTGGTAATGGAAAAGTGT-3’ and reverse primer 5’-CAAAAAATCTCAACAAACTCACACCA-3’, resulting in a PCR product of 86 base pairs. These primers have undergone extensive characterization by previous research groups [27-29]. The PCR conditions were as follows: an initial denaturation at 95°C for 10 minutes, followed by 35 cycles of denaturation at 94°C for 15 seconds, annealing at 55°C for 30 seconds, extension at 72°C for 30 seconds, and a final extension at 72°C for 10 minutes. Following amplification, 10 μL of PCR products were subsequently loaded onto 2% agarose gels that were stained with safe stain and subjected to electrophoresis. The gels were then visualized using a UV transilluminator (BIO View). The observation of a methylated band was classified as "positive" for *BRCA1 *promoter methylation.

RNA extraction and cDNA synthesis

Total RNA extraction was performed using the RNeasy FFPE kit (Cat. No. 73504, QIAGEN), following the manufacturer's specified protocol. The concentration and quality of the isolated RNA were assessed using a Nanodrop spectrophotometer (Biometrics; OneDrop TOUCH, Thermo Fisher Scientific, Waltham, Massachusetts). To determine the purity of RNA, the absorbance ratio at 260 nm to 280 nm was measured, with a requirement of being greater than 1.8. The First Strand cDNA synthesis kit (Cat. No. PR008, CANVAX, Córdoba, Spain) was utilized for cDNA synthesis. The cDNA was acquired using the Mastercycler pro-PCR System (Eppendorf, Hamburg, Germany) in thermal cycling processes. The reverse transcription procedure involved a preliminary step at 25°C for five minutes, followed by an incubation period of 45 minutes at 54°C. In addition to the tumor samples, positive control (*GAPDH*), RT-negative, and no template negative control were prepared for the purpose of reverse transcription. Given the disparity in total RNA concentrations across all samples, different quantities of RNA were employed for each sample.

Quantitative real-time PCR and RNA expression

Quantitative real-time PCR was executed using the BIO-RAD CFX96 Touch RT-PCR Machine (Bio-Rad Laboratories, Hercules, California) and RT2 SYBR Green ROX FAST Mastermix (QIAGEN GmbH, Germany) to assess the expression of *BRCA1*. The primers used for *BRCA1* were selected based on exon-exon junction, while *GAPDH* was used as an internal control for normalization (housekeeping gene). Primers used in the qPCR reaction were: 5’-ACAGCTGTGTGGTGCTTCTGTG-3’ (forward) and 5’-CATTGTCCTCTGTCCAGGCATC-3’ (reverse) for *BRCA1*; 5'-GAAGGTGAAGGTCGGAGTC-3' (forward) and 5'-GAAGATGGTGATGGGATTTC-3' (reverse) for GAPDH.

The relative qPCR was carried out in triplicate. The values were acquired as the threshold cycle (Ct) for *BRCA1* and were subsequently normalized with the housekeeping gene *GAPDH*. To determine the relative changes in gene expression for *BRCA1* in tumor and control tissue samples, the 2−ΔCt method was used. The calculations were performed separately, with the housekeeping gene *GAPDH* serving as the internal control. Based on the qPCR results, the formula 2−ΔΔCt was employed for the statistical analysis of tissue comparisons. The obtained value conveyed the expression level of the *BRCA1* gene in the PCa tumor sample in relation to the control tissue, resulting in fold change information.

Statistical analysis

Data analysis was conducted using the software GraphPad Prism 10 (GraphPad Software, Inc., San Diego, California). The comparison of methylation status and clinicopathological characteristics was conducted through chi-square tests. The two-tailed unpaired t-test was used to determine statistical differences in the relative expression data between the tumor tissue of patients with PCa (n=30) and control tissues (n=10). The calculation of the area under the curve (AUC) for *BRCA1* concentration was performed using a receiver operating characteristic curve (ROC). The median and interquartile range were used to present the data. Statistical significance was assessed at p ≤0.05 for all tests.

## Results

**Figure 1:**
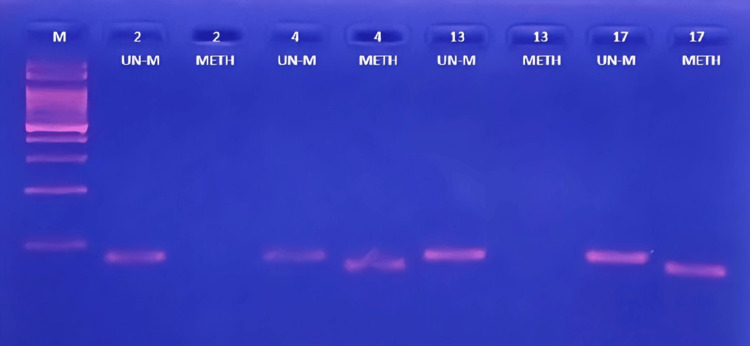
Agarose gel electrophoresis (2%) of MSP amplification products specific to the BRCA1 promoter region from bisulfite-treated DNA in human prostate tumor tissue. The first lane (M) represents a 100 bp size DNA ladder marker. Lanes 1-8 represent amplification products of unmethylated (UN-M) 86 bp or methylated (METH) 75 bp from prostate tumor samples.

The clinical characteristics of the 30 cancer patients are briefly summarized in Table 1. Within this group of patients, the median age was 70 years, with a range of 53 to 89 years. In the study group comprising PCa patients, we evaluated the promoter methylation of *BRCA1* in both cancerous and non-cancerous tissue. Out of the 30 tumors examined, methylation of the *BRCA1 *promoter was found in 76.6% (23 cases), with all 23 tumor samples exhibiting a positive methylated reaction. Conversely, no promoter hypermethylation was observed in the *BRCA1* gene among normal tissue samples. The methylation status of the *BRCA1* promoter was visualized through methylation-specific PCR, as depicted in Figure 1. There was a statistically significant disparity in methylation frequency between cancerous and normal tissue (p = 0.0001), as presented in Table 2.

**Table 1:** Clinicopathological characteristics of men with prostate cancer in the study group PSA: prostate-specific antigen.

Variable	Number of patients (%)
Total number of patients	30
Age, median (range)	70 (53–89)
50-59	2 (6.67%)
60-69	10 (33.33%)
70-79	15 (50%)
80-89	3 (10%)
PSA level, median (range)	65 (4-198)
Grade	
2	3 (10%)
3	7 (23.33%)
4	7 (23.33%)
5	13 (43.33%)
Gleason score	
7 (3+4)	3 (10%)
7 (4+3)	7 (23.33%)
8 (4+4)	7 (23.33%)
9 (4+5 and 5+4)	10 (33.33%)
10 (5+5)	3 (10%)

**Table 2:** Frequency of BRCA1 promotor methylation in cancerous and normal prostate tissue

	Tumor (n=30)	Control (n=10)	p-value
Methylated	23	0	0.0001
Unmethylated	7	10	

The association between *BRCA1 *promoter methylation and clinicopathological characteristics of prostate tumors can be observed in Table 3. The methylation status showed a significant increase in the advanced stages of PCa (grades 4 and 5) compared to lower stages (grades 2 and 3) (p = 0.01). Moreover, a significant association was discovered between *BRCA1 *promoter hypermethylation and high Gleason scores compared to low Gleason scores (p = 0.007). However, the analysis revealed no significant association between *BRCA1 *methylation status and the age of the patients.

**Table 3:** Association between BRCA1 promoter methylation and clinicopathological features of prostate cancer

Variables	Case (n=30)	*BRCA1* promoter methylation		p-value
		Positive (n=23)	Negative (n=7)	
Age (years)				
<70	12 (40%)	9 (75%)	3 (25%)	0.8
≥70	18 (60%)	14 (77.8%)	4 (22.2%)	
Grade				
2	3 (10%)	1 (33.3%)	2 (67.7%)	0.007
3	7 (23.33%)	3 (42.9%)	4 (57.1%)	
4	7 (23.33%)	6 (85.7%)	1 (14.3%)	
5	13 (43.33%)	13 (100%)	0 (0%)	
Gleason score				
7 (3+4)	3 (10%)	1 (33.3%)	2 (67.7%)	0.01
7 (4+3)	7 (23.33%)	3 (42.9%)	4 (57.1%)	
8 (4+4)	7 (23.33%)	6 (85.7%)	1 (14.3%)	
9 (4+5 and 5+4)	10 (33.33%)	10 (100%)	0 (0%)	
10 (5+5)	3 (10%)	3 (100%)	0 (0%)	

**Figure 2:**
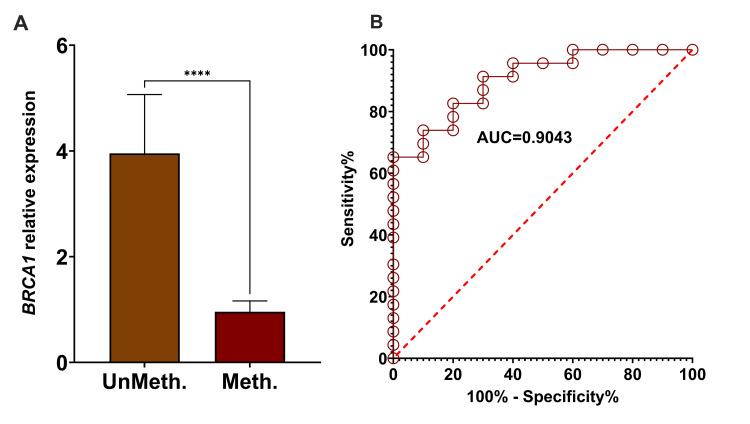
Comparison and AUC value of relative fold expression of BRCA1 in methylated and unmethylated samples. (A) The histogram shows the comparison of relative expression levels of BRCA1 between methylated tumor samples and unmethylated normal tissue. The comparison was performed using an unpaired Mann-Whitney test. (B) The AUC for BRCA1 relative expression was 0.9043. ****p<0.0001. AUC: area under the curve; PCa: prostate cancer; BRCA1: breast cancer gene 1; Meth.: methylated; UnMeth.: unmethylated.

A total of 30 prostate tumors and 10 control samples were assessed for the expression level of *BRCA1 *mRNA. The expression level of *BRCA1 *mRNA showed a significant decrease in *BRCA1 *hypermethylated patients compared to unmethylated controls (p = 0.0001), as shown in Figure 2A. The ROC curve presented in Figure 2B shows the relative expression levels of methylated and unmethylated *BRCA1*, exhibiting an area under the curve (AUC) of 0.9043, which indicates a high rate of sensitivity and specificity.

## Discussion

Prostate cancer frequently exhibits hypermethylation of genes associated with tumor suppression, DNA damage repair, cell adhesion, cell cycle regulation, apoptosis, signal transduction, and hormonal responses. Notable examples include *APC*, *p16*, *GSTP1*, *RARβ*, *CDH13*, *DAPK*, *RASSF1A*, *FHIT*, *CDH1*, and *MGMT *[30-35]. Certain genes that are hypermethylated in PCa also exhibit hypermethylation in various other cancer types. For instance, p16 demonstrates hypermethylation in colorectal cancer, leukemia, and gastric cancer, as well as *CDH1*, *APC*, and *CDH13 *in leukemia [36-41]. Methylation levels were found to be higher in samples from PCa compared to samples from benign prostatic hyperplasia (BPH) and nonmalignant tissues [31,33]. Additionally, the downregulation of tumor suppressor genes *GSTP1*, *MGMT*, and *APC *is significantly correlated with their methylation, indicating a significant involvement of DNA methylation in the advancement of carcinogenesis and disease progression [30,35]. To investigate the potential age-related occurrence of methylation, Kang et al. analyzed the methylation status of several genes (*COX2*, *APC*, *DAPK*, *GSTP1*, *CDH1*, *MGMT*, *p16*, *p14*, *RASSF1A*, *THBS1*, and *RUNX3*) in non-neoplastic prostate samples, primarily obtained from older individuals. The results indicate a significant absence of promoter methylation in these samples, suggesting that hypermethylation of specific loci in PCa is more likely a tumor-specific event rather than age-related [35].

Many researchers have employed methylation-specific PCR (MSP) to identify hypermethylation of specific regions within the CpG islands of the *BRCA1 *promoter [29,42-44]. Due to its cost-effectiveness, MSP allows for the analysis of genomic DNA derived from FFPE tissues, making it easily applicable in clinical settings. There has been no investigation into the promoter methylation of the *BRCA1* gene in PCa patients in Iraq. Therefore, this study represents the first report on hypermethylation of the *BRCA1 *gene. The observed correlations between *BRCA1 *promoter methylation and advanced cancer stage, as well as the Gleason score, suggest a potential involvement of *BRCA1 *methylation in the mechanism of tumor invasion. Moreover, a significant association was discovered between promoter hypermethylation and reduced expression of *BRCA1 *mRNA. Based on the aforementioned correlations, it is not unexpected that *BRCA1 *promoter hypermethylation could be used as a predictor of a poor outcome.

The perfect diagnostic test is commonly defined as being safe, accurate, easily obtainable, actionable, and exhibiting a favorable benefit-to-harm ratio [45]. Despite the advantageous practical features of PSA alone, the absence of cancer specificity necessitates the utilization of an additional test to enhance screening outcomes [46]. The development of DNA methylation-based biomarkers for PCa could prove beneficial, as alterations in DNA methylation patterns are among the earliest modifications observed in the progression of cancer. Therefore, the hypermethylation of the *BRCA1* gene promoter may be useful as a promising new biomarker for the detection of PCa.

It is important to acknowledge certain limitations, including a limited sample size of patients and control groups, the absence of inherited cases, insufficient clinical data such as patients' diet and lifestyle that may influence *BRCA1* promoter methylation, and the lack of post-therapy follow-up to predict recurrence and survival. Each of these factors has the potential to introduce bias into the results. However, it is crucial to mention that our study is limited to the examination of promoter methylation of the *BRCA1 *gene. Methylation within intragenic sequences may also have a crucial impact on tissue-specific gene expression.

## Conclusions

In conclusion, the utilization of the biomarker, aberrant *BRCA1 *promoter methylation, for early detection of PCa patients may prove beneficial for clinicians in adapting treatment strategies to enhance patient survival. Interestingly, our findings regarding the aberrant promoter methylation of *BRCA1 *genes exhibited a high level of statistical significance. Therefore, we propose this as a valuable biomarker strategy for the early detection of PCa.

Conducting additional analysis on the novel CpG islands of various genes could prove beneficial in developing a gene panel that can be attributed to PCa. Further research is necessary to gain a deeper understanding of the involvement of additional epigenetic mechanisms, such as micro-RNA expression or histone modification, in gene expression in PCa. Conducting research with a larger sample size could enhance the validation of biomarkers for early detection and prevention strategies for the disease.
